# Factors associated with foot ulceration and amputation in adults on dialysis: a cross-sectional observational study

**DOI:** 10.1186/s12882-017-0711-6

**Published:** 2017-09-08

**Authors:** Michelle R. Kaminski, Anita Raspovic, Lawrence P. McMahon, Katrina A. Lambert, Bircan Erbas, Peter F. Mount, Peter G. Kerr, Karl B. Landorf

**Affiliations:** 1 Discipline of Podiatry, School of Allied Health, La Trobe University, Melbourne, VIC 3086 Australia; 2 Departments of Renal Medicine & Obstetric Medicine, Eastern Health Clinical School, Monash University, Melbourne, VIC 3128 Australia; 3 College of Science, Health and Engineering, School of Psychology and Public Health, Department of Public Health, La Trobe University, Melbourne, VIC 3086 Australia; 4 Department of Nephrology, Austin Health, Melbourne, VIC 3084 Australia; 5 Department of Nephrology, Monash Health, Melbourne, VIC 3168 Australia; 6 Melbourne Health, 300 Grattan Street, Parkville, Melbourne, VIC 3050 Australia

**Keywords:** Amputation, Chronic kidney failure, Dialysis, Foot ulcer, Risk factors

## Abstract

**Background:**

Adults on dialysis are at increased risk of foot ulceration, which commonly precedes more serious lower limb complications, including amputation. Limited data exist regarding the prevalence and factors associated with foot disease in this population. Hence, this study set out to investigate factors associated with foot ulceration and amputation in a dialysis cohort.

**Methods:**

This study presents a cross-sectional analysis of baseline data from a multi-center prospective cohort study. We recruited 450 adults with end-stage renal disease on dialysis from multiple satellite and home-therapy dialysis units in Melbourne, Australia from January to December 2014. Data collection consisted of a participant interview, medical record review, health-status questionnaire and non-invasive foot examination. Logistic regression analyses were conducted to evaluate associations between screened variables and study outcomes.

**Results:**

Mean age was 67.5 (SD, 13.2) years, 64.7% were male, 94% were on hemodialysis, median dialysis duration was 36.9 (IQR, 16.6 to 70.1) months, and 50.2% had diabetes. There was a high prevalence of previous ulceration (21.6%) and amputation (10.2%), 10% had current foot ulceration, and 50% had neuropathy and/or peripheral arterial disease. Factors associated with foot ulceration were previous amputation (OR, 10.19), peripheral arterial disease (OR, 6.16) and serum albumin (OR, 0.87); whereas previous and/or current ulceration (OR, 167.24 and 7.49, respectively) and foot deformity (OR, 15.28) were associated with amputation.

**Conclusions:**

Dialysis patients have a high burden of lower limb complications. There are markedly higher risks of foot ulceration and/or amputation in those with previous and/or current ulceration, previous amputation, peripheral arterial disease, lower serum albumin, and foot deformity. Although not a major risk factor, diabetes in men was an important effect modifier for risk of ulceration.

**Electronic supplementary material:**

The online version of this article (10.1186/s12882-017-0711-6) contains supplementary material, which is available to authorized users.

## Background

Adults with chronic kidney disease (CKD), particularly those with end-stage renal disease (ESRD) requiring dialysis, have an increased risk of foot ulceration and lower extremity amputation [1–5]. As a result, dialysis patients have high rates of foot-related hospital admissions [6]. This is a serious problem due to the morbidity associated with ulceration and amputation, and the increased risk of mortality once a patient has an ulcer or amputation [1, 7–10]. There is also the associated economic burden, which is substantial [11]. For example, it has been estimated that the direct cost of healing one infected foot ulcer (without amputation) is US$17,500 [12].

We recently conducted a systematic review [13] and found prevalence estimates of 14.4% for foot ulceration and 5.9% for amputation in adults on dialysis. Our meta-analysis showed that ulceration and/or amputation were associated with male sex, current smoking, diabetes mellitus (increasing with longer duration), retinopathy, coronary artery disease, elevated serum phosphorus and glycated hemoglobin, lower serum albumin, previous ulceration or amputation, peripheral arterial disease and neuropathy [13].

In light of these findings, there appears to be a strong link between ESRD and risk of ulceration and amputation. At present, the central determinants contributing to the development of these conditions are not fully understood. Existing cross-sectional studies often lack adequate sample sizes, do not encompass a broad range of risk factors, include only those with diabetes, and have focused on risk factors for amputation. Given the limitations of current evidence and the high impact of these foot problems, the aim of this study was to investigate the prevalence and factors associated with foot ulceration and amputation in adults with ESRD on dialysis.

## Methods

### Study design

Data investigated in this study are baseline data from a prospective cohort study – detailed inclusion criteria, recruitment and methods are published elsewhere [14]. Here we describe participant characteristics at baseline and conduct a cross-sectional analysis of the prevalence of lower limb complications and factors associated with foot ulceration and amputation.

### Participants

**Fig. 1:**
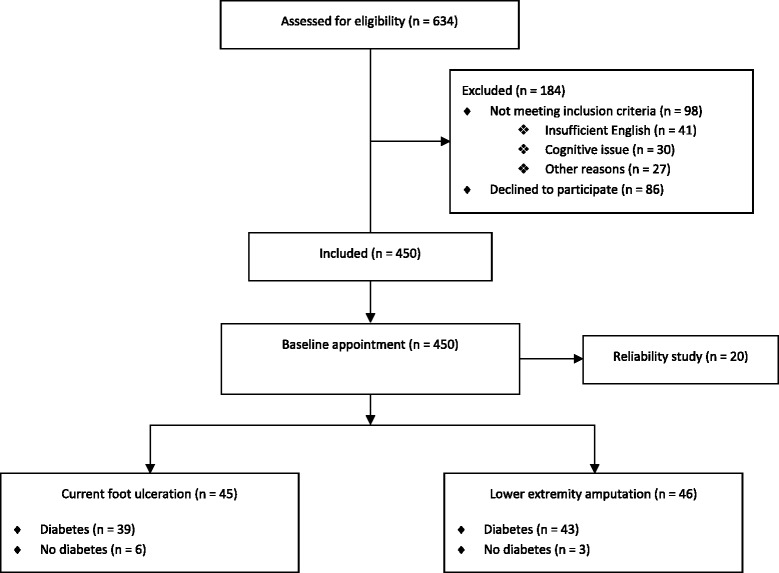
Flow of study participants

Between January and December 2014, 450 patients were recruited from satellite and home-therapy dialysis units across multiple health organizations in Melbourne, Australia. Participants were eligible if they had ESRD and were clinically stable on dialysis (hemodialysis or peritoneal dialysis), aged 18 years or over, and able to provide informed consent. Exclusion criteria included insufficient English language skills to provide informed consent or follow instructions. Figure 1 outlines the flow of participants for this study.

### Outcomes

Outcomes of interest included ‘current foot ulceration’ and ‘lower extremity amputation’. Foot ulceration was defined as a ‘full thickness skin break distal to the ankle joint, which may extend into or through the dermis and involve deeper structures such as bones, tendons, joint capsules and ligaments’ [15–17]. Amputation was defined as a ‘complete loss of any part of the lower extremity [18], including any digit, partial foot amputation or higher’. Amputations resulting from accidental trauma (unrelated to ESRD) or the presence of a tumor were not recorded. A major amputation was documented if there was loss of a limb above the ankle and a minor amputation if below the ankle.

### Data collection

Data collection consisted of a participant interview, medical record review, health-status questionnaire and non-invasive foot assessment. One examiner (M.R.K.) collected the data and performed all foot assessments. Details of the participant interview, medical record review, and other methods, including validity and reliability, have been previously described [14].

Peripheral neuropathy was defined as known neuropathy documented in the medical records, monofilament score < 3/3 over the plantar aspects of the hallux, first and fifth metatarsals and/or vibration perception threshold >25 V over the apex of the hallux. Peripheral arterial disease was defined as known peripheral arterial disease and/or history of lower extremity revascularization procedure documented in the medical records, absence of ≥2 pedal pulses (i.e. dorsalis pedis and posterior tibial), toe-brachial pressure index ≤0.6, and/or ankle-brachial pressure index ≤0.9. Arterial calcification was defined as an ankle-brachial pressure index >1.3 and/or non-compressible peripheral arteries (i.e. systolic pressure >240 mmHg).

Foot deformity was assessed visually and defined as any of the following: hallux abducto valgus, hammer/claw toes, bony prominences (e.g. prominent metatarsal heads), Charcot foot, and other. Limited first metatarsophalangeal joint (MTPJ) range of motion was defined as <65° passive, non-weightbearing dorsiflexion [19]. Plantar pressures were evaluated using a two-step gait initiation protocol [20] with the Tekscan Matscan® system (Tekscan Inc., South Boston, MA, USA). Peak plantar pressures were measured for the total left and right foot [20] and the mean of three values (kg/cm^2^) was used for analysis.

Skin and nail pathologies were assessed visually, as previously described [14]. Inappropriate footwear was defined as poor shoe fit, inappropriate shoe style and/or poor shoe condition [21]. Foot-health care behaviors were self-reported according to a series of five questions [14]. Foot-health care behavior was considered ‘poor’ if participants answered ‘no’ to three or more questions. Regular podiatry attendance (i.e. number of attendances in the last 12 months) was determined by self-report.

Generic health status was assessed using the Short-Form 36 version 2.0 health survey (SF-36v2®) [22–25].

### Reliability

To ensure reliability of the foot assessments, 20 participants were assessed on two separate occasions spaced 1 week apart by the same blinded examiner (M.R.K.). Details for intra-examiner reliability have been described elsewhere [14], and results are shown in Additional file 1. Briefly, intra-class correlation coefficients ranged from 0.87 to 0.99 for continuous data and all weighted kappa values equaled 1.00 with the absolute percentage of agreement ranging from 95 to 100% for dichotomous data.

### Statistical analysis

Participant characteristic and health-related quality of life data were calculated and expressed as mean (SD) for normally distributed data or median (IQR) if not normally distributed. Continuous data were checked for normality using the Shapiro-Wilks and Kolmogorov-Smirnov tests, and by assessing skewness and kurtosis values. Independent samples *t*-tests were calculated to compare mean differences between groups (e.g. foot ulcer or no foot ulcer), while Mann-Whitney *U* tests were calculated for non-normally distributed data. For categorical variables, n (%) were recorded and Chi-square (χ^2^) tests calculated to explore between-groups differences.

Univariate and multivariate logistic regression analyses were undertaken to evaluate associations between screened variables and outcomes (i.e. foot ulceration and amputation). For each outcome, base regression models were developed to fit each factor, one at a time, with other variables including participant characteristics and possible confounders (Additional file 2) and statistical significance was assessed. As a sensitivity analysis, forward selection was used when building models with *P*-values set at 0.1 [14]. Confounders were retained if they changed the estimated association between risk factors and the outcome by 10% or more, or were significant at the 5% level in adjusted models [26].

Possible interactions were also assessed. We set a significance value of p-interaction at <0.1 so that we did not miss any important interactions (i.e. as the statistical power to test for significant interaction is lower than to test for the main effect) [27]. Strata specific analyses of regression models using diabetes (yes/no) were conducted to assess variables for possible effect modification and if there was evidence of effect modification in models with an interaction term. Risk estimates were presented as odds ratios (ORs) with corresponding 95% confidence intervals (CIs).

As a consequence of only half the sample having coexisting diabetes, the variable ‘diabetes duration’ was removed completely from the multivariate logistic regression analyses due to the substantial number of missing cases (*n* = 224). Similarly, the variables ‘glycated hemoglobin’ and ‘peak plantar pressure (left and right foot)’ had missing data in 8.7% and 12.2% of the sample, respectively. No significant or confounding effects from these three variables, individually or combined, were found after removal of missing cases. Finally, as limited first MTPJ range of motion was present in the majority of the sample (93.6%) and in all participants with current foot ulceration or amputation, this variable was also removed from the logistic regression analyses.

Data analysis was undertaken using IBM SPSS version 23.0 (IBM Corp, Somers, NY, USA), Stata 11 Data Analysis and Statistical Software (StataCorp LP, Texas, USA), QualityMetric Health Outcomes™ Scoring Software 4.5.1, and FootMat™ 7.0 Software (Tekscan Inc., South Boston, MA, USA).

## Results

We recruited 450 adults with ESRD treated with dialysis. The mean age (SD) was 67.5 (13.2) years, 64.7% were male, and the mean body mass index (SD) was 28.2 (6.6) kg/m^2^. The majority were treated with hemodialysis (94%) compared to peritoneal dialysis (6%). Median duration of dialysis was 36.9 (IQR, 16.6 to 70.1) months. Half the participants had coexisting diabetes (50.2%), with a mean diabetes duration (SD) of 256.3 (152.6) months. Physical component scores in the SF-36v2 were significantly lower in those with current ulceration and/or amputation, compared to those with no foot complications (*p* = 0.001). Participant characteristics, comorbidities, laboratory values and health-related quality of life scores for those with/without current foot ulceration and/or amputation are shown in Table 1.

**Table 1:** Participant characteristics – data are n (%), unless otherwise stated

		Current foot ulceration			Lower extremity amputation		
	Total (*N* = 450)	Yes (*n* = 45)	No (*n* = 405)	*P*-value*	Yes (*n* = 46)	No (*n* = 404)	*P*-value*
Mean age (SD), *years*	67.5 (13.2)	69.0 (10.3)	67.3 (13.5)	0.33	65.4 (11.2)	67.7 (13.4)	0.25
Male sex	291 (64.7)	36 (80.0)	255 (63.0)	0.035*	37 (80.4)	254 (62.9)	0.028*
Mean body mass index (SD), *kg/m* ^*2*^	28.2 (6.6)	29.1 (6.5)	28.1 (6.6)	0.30	30.4 (6.3)	27.9 (6.6)	0.014*
Current smoking	54 (12.0)	5 (11.1)	49 (12.1)	1.00	5 (10.9)	49 (12.1)	0.99
Living alone	75 (16.7)	6 (13.3)	69 (17.0)	0.67	10 (21.7)	65 (16.1)	0.44
Ethnicity							
Indigenous Australian	4 (0.9)	0 (0)	4 (1.0)	1.00	1 (2.2)	3 (0.7)	0.88
English	85 (18.9)	9 (20)	76 (18.8)	1.00	12 (26.1)	73 (18.1)	0.94
European	194 (43.1)	16 (35.6)	178 (44.0)	0.36	14 (30.4)	180 (44.6)	0.60
American	4 (0.9)	0 (0)	4 (1.0)	1.00	0 (0)	4 (1.0)	1.00
African	14 (3.1)	1 (2.2)	13 (3.2)	1.00	2 (4.3)	12 (3.0)	0.95
Asian	67 (14.9)	5 (11.1)	62 (15.3)	0.60	1 (2.2)	66 (16.3)	0.88
Pacific Islander	29 (6.4)	3 (6.7)	26 (6.4)	1.00	5 (10.9)	24 (5.9)	0.33
Other^a^	53 (11.8)	11 (24.4)	42 (10.4)	0.011*	11 (23.9)	42 (10.4)	0.159
Cause of ESRD							
Diabetes mellitus	180 (40.0)	37 (82.2)	143 (35.3)	<0.001*	42 (91.3)	138 (34.2)	0.98
Hypertension	28 (6.2)	1 (2.2)	27 (6.7)	0.40	1 (2.2)	27 (6.7)	0.82
Glomerulonephritis	97 (21.6)	2 (4.4)	95 (23.5)	0.006*	0 (0)	97 (24.0)	0.88
Polycystic kidney disease	22 (4.9)	1 (2.2)	21 (5.2)	0.61	1 (2.2)	21 (5.2)	0.104
Reflux	19 (4.2)	1 (2.2)	18 (4.4)	0.76	1 (2.2)	18 (4.5)	0.67
Renovascular disease	10 (2.2)	0 (0)	10 (2.5)	0.59	1 (2.2)	9 (2.2)	0.58
Vasculitis	9 (2.0)	0 (0)	9 (2.2)	0.65	0 (0)	9 (2.2)	0.64
Unknown	15 (3.3)	1 (2.2)	14 (3.5)	1.00	0 (0)	15 (3.7)	0.37
Other	70 (15.6)	2 (4.4)	68 (16.8)	0.05	0 (0)	70 (17.3)	0.88
Dialysis treatment							
Hemodialysis	423 (94.0)	42 (93.3)	381 (94.1)	1.00	43 (93.5)	380 (94.1)	0.41
Peritoneal dialysis							
CAPD	9 (2.0)	0 (0)	9 (2.2)	0.65	0 (0)	9 (2.2)	0.64
APD	18 (4.0)	3 (6.7)	15 (3.7)	0.58	3 (6.5)	15 (3.7)	0.79
Median duration of dialysis (IQR), *months*	36.9 (16.6 to 70.1)	37.5 (20.0 to 64.1)	36.8 (16.5 to 71.8)	0.91	38.3 (17.7 to 72.6)	36.6 (16.6 to 69.5)	0.77
Diabetes mellitus	226 (50.2)	39 (86.7)	187 (46.2)	<0.001*	43 (93.5)	183 (45.3)	<0.001*
Type 1	13 (5.8)	4 (10.3)	9 (4.8)	0.34	7 (15.2)	6 (1.5)	0.003*
Type 2	213 (94.2)	35 (89.7)	178 (95.2)	0.34	36 (78.3)	177 (43.8)	0.003*
Mean duration (SD), *months*	256.3 (152.6)	348.8 (167.6)	237.0 (142.3)	<0.001*	301.8 (163.1)	245.6 (148.5)	0.043*
Retinopathy	132 (29.3)	28 (62.2)	104 (25.7)	<0.001*	32 (69.6)	100 (24.8)	<0.001*
Known peripheral neuropathy^b^	70 (15.6)	20 (44.4)	50 (12.3)	<0.001*	26 (56.5)	44 (10.9)	<0.001*
Known peripheral arterial disease^b^	79 (17.6)	27 (60.0)	52 (12.8)	<0.001*	31 (67.4)	48 (11.9)	<0.001*
Lower extremity revascularization procedure	35 (7.8)	22 (48.9)	13 (3.2)	<0.001*	20 (43.5)	15 (3.7)	<0.001*
Hypertension (requiring medication)	360 (80.0)	36 (80.0)	324 (80.0)	1.00	40 (87.0)	320 (79.2)	0.29
Dyslipidemia	301 (66.9)	38 (84.4)	263 (64.9)	0.013*	39 (84.8)	262 (64.9)	0.011*
Ischemic heart disease	263 (58.4)	36 (80.0)	227 (56.0)	0.003*	33 (71.7)	230 (56.9)	0.076
Congestive cardiac failure	122 (27.1)	18 (40.0)	104 (25.7)	0.061	15 (32.6)	107 (26.5)	0.48
Cerebrovascular disease	104 (23.1)	19 (42.2)	85 (21.0)	0.003*	15 (32.6)	89 (22.0)	0.153
Osteoarthritis	192 (42.7)	17 (37.8)	175 (43.2)	0.60	15 (32.6)	177 (43.8)	0.194
Inflammatory arthritis	183 (40.7)	14 (31.1)	169 (41.7)	0.22	13 (28.3)	170 (42.1)	0.099
Median C-reactive protein (SD), *mg/L* ^c,d^	7.33 (2.83 to 19.67)	10.00 (4.82 to 43.83)	6.95 (2.67 to 18.08)	0.011*	10.89 (4.44 to 36.88)	6.90 (2.67 to 18.50)	0.026*
Mean serum albumin (SD), *g/L* ^c^	33.73 (3.94)	31.12 (4.76)	34.01 (3.74)	<0.001*	32.50 (4.32)	33.86 (3.88)	0.045
Mean total calcium (SD), *mmol/L* ^c^	2.20 (0.14)	2.15 (0.13)	2.20 (0.14)	0.008*	2.21 (0.12)	2.20 (0.14)	0.69
Mean phosphate (SD), *mmol/L* ^c^	1.55 (0.38)	1.64 (0.39)	1.54 (0.38)	0.135	1.59 (0.41)	1.55 (0.38)	0.53
Median parathyroid hormone (SD), *pmol/L* ^c^	29.58 (18.04 to 45.84)	28.03 (20.70 to 43.02)	29.77 (17.38 to 46.25)	0.83	28.52 (21.56 to 50.24)	29.65 (17.38 to 45.43)	0.54
Mean glycated hemoglobin (SD), *%* ^c,d^	6.14 (1.31)	6.89 (1.24)	6.05 (1.29)	<0.001*	6.98 (1.36)	6.03 (1.27)	<0.001*
Median hemoglobin (IQR), *g/L* ^c^	111.33 (102.92 to 117.67)	108.00 (99.50 to 115.83)	111.67 (103.67 to 117.67)	0.060	108.50 (99.25 to 115.25)	111.67 (103.67 to 117.67)	0.080
Mean SF-36v2® PCS (SD)	38.14 (10.70)	33.08 (8.92)	38.71 (10.74)	0.001*	33.16 (9.93)	38.71 (10.65)	0.001*
Mean SF-36v2® MCS (SD)	48.55 (11.40)	47.08 (13.96)	48.71 (11.09)	0.45	47.11 (13.56)	48.71 (11.14)	0.44
Previous foot ulceration	97 (21.6)	32 (71.1)	65 (16.0)	<0.001*	44 (95.7)	53 (13.1)	<0.001*
Current foot ulceration	45 (10.0)	45 (100.0)	N/A	N/A	26 (56.5)	19 (4.7)	<0.001*
Lower extremity amputation	46 (10.2)	26 (57.8)	20 (4.9)	<0.001*	46 (100)	N/A	N/A
Minor	39 (8.7)	22 (48.9)	17 (4.2)	<0.001*	39 (84.8)		
Major	12 (2.7)	8 (17.8)	4 (1.0)	<0.001*	12 (26.1)		
Combination	5 (1.1)	4 (8.9)	1 (0.2)	<0.001*	5 (10.9)		

*Significant difference between ‘foot ulceration/amputation’ and ‘no foot ulceration/amputation’ groups, *p* < 0.05. *SD* Standard deviation. ^a^‘Ethnicity other’ were for participants that identified themselves as ‘Australian’ (excluding Indigenous Australians). *ESRD* End-stage renal disease. *CAPD* Continuous ambulatory peritoneal dialysis. *APD* Automated peritoneal dialysis. *IQR* Interquartile range. ^b^Known peripheral neuropathy/known peripheral arterial disease were defined as a history of peripheral neuropathy/peripheral arterial disease documented in the medical records. ^c^An average of the three latest laboratory test values was collected. ^d^Maximum missing data were for glycated hemoglobin involving 39 participants overall (8.7%). Missing data were for glycated hemoglobin (*n* = 39) and C-reactive protein (n = 3). SF-36v2® = Short-Form-36 version 2.0. *PCS* Physical component score. *MCS* Mental component score. *N/A* Not applicable

Overall, there was a high prevalence of previous foot ulceration (21.6%), current foot ulceration (10.0%), and lower extremity amputation (10.2%). The total number of foot ulcers was 68. Current foot ulcers were predominantly neuro-ischemic (69.1%), located on the dorsal, medial or lateral toes (52.9%) and had a median duration of 3.0 (IQR, 1.2 to 6.0) months (Additional file 3). There were a total of 79 amputations, most of which were minor (83.5%), such as a toe (Additional file 3). Additional file 4 provides comparisons of identified foot problems according to diabetes status for those with/without ulceration and amputation.

Foot examination, foot-health care behaviors and podiatry attendance results are outlined in Table 2. Half the participants had peripheral neuropathy (50.7%) and/or peripheral arterial disease (52.4%). There was a high prevalence of arterial calcification (40.9%), limited first MTPJ range of motion (93.6%), foot deformity (75.8%), and skin and nail pathology (87.8% and 70.9%, respectively). Peak plantar pressures (kg/cm^2^) were significantly higher in those with ulceration (left mean difference [MD], 0.22 [CI, 0.00 to 0.43], right MD, 0.28 [CI, 0.06 to 0.50]) or amputation (left MD, 0.22 [CI, 0.05 to 0.38], right MD, 0.27 [CI, 0.06 to 0.49]), compared to those without these conditions. A large proportion of participants presented with inappropriate/ill-fitting footwear (66.0%), almost one third had poor foot-health care behaviors (30.2%), and only half had seen a podiatrist in the last year (49.6%). Additional file 5 presents health-related quality of life (SF-36v2®), foot examination and foot-health care behavior results.

**Table 2:** Foot examination, foot-health care behaviors and podiatry attendance – data are n (%), unless otherwise specified

		Foot ulceration			Lower extremity amputation		
	Total (N = 450)	Yes (n = 45)	No (n = 405)	*P*-value*	Yes (n = 46)	No (n = 404)	*P*-value*
Peripheral neuropathy^a^	228 (50.7)	43 (95.6)	185 (45.7)	<0.001*	44 (95.7)	184 (45.5)	<0.001*
Peripheral arterial disease^b^	236 (52.4)	42 (93.3)	194 (47.9)	<0.001*	39 (84.8)	197 (48.8)	<0.001*
Arterial calcification	184 (40.9)	20 (44.4)	164 (40.5)	0.73	21 (45.7)	163 (40.3)	0.592
Foot deformity	341 (75.8)	39 (86.7)	302 (74.6)	0.107	42 (91.3)	299 (74.0)	0.016*
Limited range of motion of first MTPJ^c^	421 (93.6)	39 (86.7)	382 (94.3)	0.29	38 (82.6)	383 (94.8)	0.30
Median peak plantar pressure (IQR), *kg/cm* ^*2*c^							
Total left foot^d^	1.74 (1.50 to 2.06)	2.00 (1.74 to 2.40)	1.73 (1.50 to 2.04)	0.007*	2.02 (1.76 to 2.36)	1.73 (1.50 to 2.03)	0.002*
Total right foot^d^	1.72 (1.50 to 2.09)	2.11 (1.68 to 2.42)	1.71 (1.49 to 2.05)	0.002*	2.09 (1.80 to 2.42)	1.71 (1.49 to 2.05)	0.001*
Skin pathology	395 (87.8)	42 (93.3)	353 (87.2)	0.34	43 (93.5)	352 (87.1)	0.31
Nail pathology	319 (70.9)	37 (82.2)	282 (69.6)	0.112	34 (73.9)	285 (70.5)	0.76
Inappropriate footwear	297 (66.0)	25 (55.6)	272 (67.2)	0.164	21 (45.7)	276 (68.3)	0.004*
Poor foot-health care	136 (30.2)	10 (22.2)	126 (31.1)	0.289	7 (15.2)	129 (31.9)	0.030*
Podiatry attendance, *last 12 months*	223 (49.6)	36 (80.0)	187 (46.2)	<0.001*	36 (78.3)	187 (46.3)	<0.001*

*Significant difference between ‘foot ulceration/amputation’ and ‘no foot ulceration/amputation’ groups, *p* < 0.05. *MTPJ* Metatarsophalangeal joint. ^a^Peripheral neuropathy was defined as documentation of known peripheral neuropathy in the medical records; monofilament score < 3/3 (either foot); and/or vibration perception threshold >25 V (either foot). ^b^Peripheral arterial disease was defined as documentation of known peripheral arterial disease in the medical records and/or history of lower extremity revascularization procedure; absence of ≥2 pedal pulses; toe-brachial pressure index ≤0.6 (either foot); and/or ankle-brachial pressure index ≤0.9. ^c^Maximum missing data were for left peak plantar pressure involving 56 participants overall (12.4%). Missing data were for limited range of motion of first MTPJ (left, *n* = 25; right, *n* = 15) and peak plantar pressures (left, *n* = 56; right, *n* = 55). ^d^Data presented are of the total foot and do not include any specific regions (e.g. hallux, 1st MTPJ, etc.). *IQR* Interquartile range

There were numerous significant risk factors associated with ulceration and amputation in the univariate analyses (Additional file 6). The multivariate logistic regression analyses for the foot ulceration and amputation models are shown in Tables 3 and 4, respectively. In the multivariate analyses, risk factors associated with foot ulceration included previous amputation (OR, 10.19 [CI, 3.14 to 33.07]), peripheral arterial disease (OR, 6.16 [CI, 1.47 to 25.80]), and serum albumin (OR, 0.87 [CI, 0.78 to 0.96]). Risk factors associated with amputation were previous foot ulceration (OR, 167.24 [CI, 23.22 to 1204.49]), current foot ulceration (OR, 7.49 [CI, 1.89 to 29.69]), and foot deformity (OR, 15.28 [CI, 2.23 to 104.55]).

**Table 3:** Multivariate logistic regression analysis for factors associated with foot ulceration – data are n (%), unless otherwise specified

	Foot ulceration		Multivariate logistic regression analysis		
Risk Factor	Yes	No			
*n*	45	405	aOR	95% CI	*P*-value*
Previous amputation	26 (57.8)	20 (4.9)	10.19	3.14 to 33.07	<0.001*
Peripheral arterial disease	42 (93.3)	194 (47.9)	6.16	1.47 to 25.80	0.013*
Mean serum albumin (SD), *g/L*	31.12 (4.76)	34.01 (3.74)	0.87	0.78 to 0.96	0.008*
Mean total calcium (SD), *mmol/L*	2.15 (0.13)	2.20 (0.14)	0.05	0.00 to 1.41	0.080
Peripheral neuropathy	43 (95.6)	185 (45.7)	5.02	0.97 to 26.02	0.055

*aOR* Adjusted odds ratio. *CI* Confidence interval. *Significant association, *p* < 0.05. *SD* Standard deviation. The final multivariate model controlled for the following confounding variables: age, sex, living alone, dialysis duration, diabetes mellitus, serum phosphate, previous foot ulceration and podiatry attendance

**Table 4:** Multivariate logistic regression analysis for factors associated with amputation – data are n (%), unless otherwise specified

	Lower extremity amputation		Multivariate logistic regression analysis		
Risk Factor	Yes	No			
*n*	46	404	aOR	95% CI	*P*-value*
Previous foot ulceration	44 (95.7)	53 (13.1)	167.24	23.22 to 1204.49	<0.001*
Current foot ulceration	26 (56.5)	19 (4.7)	7.49	1.89 to 29.69	0.004*
Foot deformity	42 (91.3)	299 (74.0)	15.28	2.23 to 104.55	0.005*
Diabetes mellitus	43 (93.5)	183 (45.3)	5.28	0.22 to 129.47	0.31
Osteoarthritis	15 (32.6)	177 (43.8)	0.26	0.07 to 1.03	0.055

*aOR* Adjusted odds ratio. *CI* Confidence interval. *Significant association, *p* < 0.05. The final multivariate model controlled for the following confounding variables: age, sex, physical component score, dialysis duration, retinopathy, ischemic heart disease, C-reactive protein, serum albumin, serum phosphate, glycated hemoglobin, peripheral neuropathy, peripheral arterial disease, inappropriate/ill-fitting footwear, nail pathology, and podiatry attendance

Although the presence of diabetes was a significant risk factor in the univariate analyses, when we entered previous amputation in the multivariable regression model for foot ulceration it became non-significant (OR, 2.13 [CI, 0.71 to 6.36]). Likewise, with amputation as the outcome, after entering previous foot ulceration in the regression model, diabetes became non-significant (OR, 4.04 [CI, 0.72 to 22.78]).

The interaction term for being male with diabetes increased the odds of foot ulceration (OR, 3.40 [CI, 1.12 to 10.33]; *p* = 0.07 from the interaction term), but not for amputation (OR, 0.96 [CI, 0.18 to 5.12]; *p* = 0.40 from the interaction term).

## Discussion

In our dialysis cohort, we found high rates of previous (21.6%) and current foot ulceration (10.0%), with comparable amputation rates (10.2%). For ulceration, the odds increased if participants had a history of amputation or peripheral arterial disease, and fell as serum albumin increased. For amputation, the odds were greater if participants had a previous and/or current ulceration, or had a foot deformity.

These findings are consistent with results from previous studies [13, 28–30]. A cross-sectional study of 466 participants with diabetes and Stage 4 or 5 CKD in the UK and US found that previous ulceration was significantly associated with amputation (OR, 42 [95% CI, 17 to 100]) [28]. A retrospective study of 218 dialysis patients in Australia also found that the odds of amputation were greater in those with previous ulceration (OR, 12.41 [95% CI, 2.23 to 69.04]) [29]. That patients with a history of amputation are more likely to have foot ulcers and vice versa is not surprising, given that one commonly precedes the other, and they often have similar ethology [13].

Our finding that those with peripheral arterial disease and hypoalbuminemia are at greater risk of ulceration is supported by previous studies [13, 28, 30, 31]. Peripheral arterial disease is detrimental to peripheral perfusion, skin integrity and ulcer healing [32]. Given that a high proportion of foot ulcers were located on the toes (52.9%), this may be reflective of the high rate of peripheral arterial disease (52.4%) identified in this dialysis sample. Low serum albumin reflects poor nutrition and/or systemic inflammation. When combined with peripheral arterial disease, there is increased risk of ulceration, wound deterioration, and predisposition to infection [32].

In our earlier meta-analysis [13] we did not find foot deformity to be a significant risk factor for amputation. This may be explained by the heterogeneity between studies and that very few studies [2, 4, 33] have assessed this variable as a risk factor. However, a recent study (published after our meta-analysis) found similarly to our current cross-sectional study that foot deformity provided more than 7.5-fold risk of amputation (CI, 1.05 to 53.86) [29].

There were three additional important findings from our study. First, diabetes did not prove to be a significant risk factor for foot ulceration or amputation in our multivariate models, despite previous claims [34–41]. One explanation for this is that other risk factors in our models, such as previous ulceration and amputation, had a stronger association with these outcomes than diabetes. Existing studies [34–41] did not include these risk factors in their analyses, therefore it is important to question whether the individual effect of diabetes on risk of ulceration or amputation may have been previously overestimated. Indeed, our findings suggest that the presence of diabetes is not the primary risk factor for ulceration or amputation in the dialysis population. However, its effect on other interrelated factors remains relevant, as diabetes was found to be a strong effect modifier with sex (particularly being male) in our ulceration model. That being said, in order to develop a history of foot disease in the first instance, other clinical risk factors (such as diabetes, neuropathy, foot deformity or peripheral arterial disease) are likely to contribute to the development of the original ulcer or amputation.

The second important finding was a high frequency of foot problems, including peripheral neuropathy, peripheral arterial disease, arterial calcification, limited first MTPJ range of motion, foot deformity, and skin and nail pathology throughout the cohort. Peripheral neuropathy and peripheral arterial disease were found to be highly prevalent in 50.7% and 52.4% of the sample, respectively, which is similar to existing studies [4, 42]. Alarmingly, only 15.6% and 17.6% of participants had neuropathy or peripheral arterial disease, respectively, documented in their medical records prior to the baseline assessment, which provides further impetus for regular foot examination in the dialysis population. Interestingly, more than one third of participants without diabetes were found to have neuropathy (35.3%). Our previous meta-analysis [13] found neuropathy to be a significant risk factor for ulceration; however, in the present study it had borderline significance (*p* = 0.055). This can be explained by the multivariate analysis combining neuropathy with other stronger factors, such as previous amputation and peripheral arterial disease, which may have confounded the effect of neuropathy. In contrast, previous cross-sectional studies have found neuropathy to be a risk factor [30, 31, 42], which highlights that this factor should not be discounted. Prospective studies are required to address this issue.

The third important finding was that there were high rates of inappropriate or ill-fitting footwear, poor foot-health care behaviors, and only half the sample had seen a podiatrist in the last 12 months. Rubbing and repetitive skin trauma are frequently caused by ill-fitting or inappropriate footwear [16]. When combined with poor foot-health care behavior (e.g. not inspecting neuropathic feet) and poor podiatry attendance, the risk of ulceration and subsequent lower limb complications, such as infection and amputation, are increased [43–45]. Although we did not find these factors to be significant, their potential involvement in ulcer formation cannot be underestimated so these factors also require investigation in prospective studies.

This study has some limitations. The true prevalence of ulceration and amputation may have been underestimated as many patients with obvious foot complications declined to participate. Minor and major amputations were combined in the analysis – due to too few cases of major amputation (*n* = 12). Therefore, an analysis separating minor and major amputations was not possible for this reason. Recall bias may have been present in this study; for example, when the participants self-reported their annual podiatry attendance rate. The examiner was also unable to collect data for some of the screened variables. Reasons for this included: unable to perform the foot or plantar pressure assessments due to amputation, Charcot foot, or wheelchair dependent, or a blood test not performed or results were unavailable. Such missing data may have affected the precision of our findings and limited the power of the study to detect other important associations with ulceration and amputation; although, given the large sample size, we believe this would be minor.

The sample was largely recruited from satellite dialysis units and the majority of participants were undertaking hemodialysis treatment, so our findings are generalizable to these patients. It is also unclear whether dialysis modality or different dialysis treatment regimens had an effect on the assessment of risk factors in our study. Previous small studies have indicated that cutaneous microcirculation may be affected during dialysis treatment [46, 47]. Although the examiner made every attempt to perform the baseline foot assessment on participants prior to dialysis or on a non-dialysis day, the majority of arterial assessments were performed on participants during their dialysis treatment. Therefore, it is uncertain whether the presence of peripheral arterial disease may have been overestimated, particularly when conducting the toe- and ankle-brachial pressure indices. Last, although footwear characteristics were assessed with a validated tool [21], the assessment was made on shoes worn by each participant to their baseline appointment, which may not have been representative. Nevertheless, patients spend a substantial time in these shoes when attending dialysis (approximately 15 h per week), so assessment of footwear worn to these appointments was deemed important.

There are also several strengths of this study. The data collection form, published elsewhere [14], was based on our systematic review [13] and constructed from a comprehensive review of the diabetes literature. The assessment tools used in the foot examination were chosen based on sound validity and reliability. Furthermore, data collection was standardized [14] and performed by one examiner (M.R.K.) to ensure consistency, thus reducing the chance of systematic error. Indeed, intra-examiner reliability was assessed and found to be excellent for the foot assessments. The study was undertaken across multiple centers and was designed to encompass a full range of risk factors to ensure generalizability to clinical practice.

Dialysis patients with ulceration have a poor prognosis for foot salvage, therefore these patients have a higher risk of amputation and foot-related mortality [7, 48–50]. Previous retrospective studies [3, 51] have found that a temporal association exists between the onset of dialysis and foot ulcer development. Early identification of those at greatest risk is essential for the prevention and management of foot complications and may improve outcomes. High-quality prospective studies are now needed to confirm the findings from this study, establish if a temporal relationship exists between these factors and foot ulceration or amputation, and to evaluate interventions that are designed to reduce the risk of foot ulceration and amputation in the dialysis population.

Our study highlights a clear need for foot care provision to dialysis patients. Untested but logical sequelae of these findings include the need for regular foot screening and assessment; to identify potential foot complications and those at the highest risk. It may also be important that patients receive comprehensive foot care education including strategies to prevent foot complications (e.g. daily foot inspection), regular podiatry consultation to ensure optimal foot health, and/or early referral to a multidisciplinary foot-care team for the management of serious foot complications. Preliminary intervention studies that have evaluated the effectiveness of foot care prevention programs for reducing ulceration and amputation in dialysis patients have shown promising results [52–57], but are often limited by small sample sizes, high attrition rate, non-random allocation of participants, lack of blinding of participants/assessors, and selection/sampling bias. As such, high-quality randomized trials are clearly required to evaluate the effectiveness of these proposed interventions.

## Conclusions

This study found that adults on dialysis have a high prevalence of foot ulceration and amputation. Dialysis patients with markedly higher risks of foot ulceration and/or amputation include those with previous or current ulceration, past amputation, peripheral arterial disease, lower serum albumin, and foot deformity. Although not a major risk factor, diabetes in men was an important effect modifier for risk of ulceration.

## Additional files

Additional file 1: Intra-examiner reliability. Tables showing the results of the intra-examiner reliability testing for dichotomous and continuous variables. (PDF 352 kb)

Additional file 2: Risk factors and potential confounding variables. A table showing the risk factors and potential confounding variables that were considered in the regression models. (PDF 361 kb)

Additional file 3: Characteristics of current foot ulcers and amputations. Tables showing the characteristics of current foot ulcers and amputations at baseline. (PDF 527 kb)

Additional file 4: Comparisons between participants with and without diabetes. Tables showing comparisons between participants with and without diabetes for the presence and absence of foot complications. (PDF 690 kb)

Additional file 5: Individual health-related quality of life, foot assessment, and foot-health care behavior results for participants with and without foot ulceration and/or amputation. Tables showing comparisons between participants with and without foot ulceration and/or amputation for health-related quality of life, foot assessment, and foot-health care behavior variables – data are presented in its entirety before the data were categorized for statistical analysis. (PDF 732 kb)

Additional file 6: Univariate analyses for factors associated with foot ulceration and lower extremity amputation. Tables showing the results of the univariate analyses for factors associated with foot ulceration and lower extremity amputation. (PDF 711 kb)

## Abbreviations

CI: Confidence interval
CKD: Chronic kidney disease
ESRD: End-stage renal disease
IQR: Interquartile range
MD: Mean difference
MTPJ: Metatarsophalangeal joint
OR: Odds ratio
SD: Standard deviation
SF-36v2: Short-Form 36 version 2.0
UK: United Kingdom
US: United States
