# Supplementary material for: Factors associated with foot ulceration and amputation in adults on dialysis: a cross-sectional observational study
Source: BMC Nephrol. 2017 Sep 8;18:293. doi: 10.1186/s12882-017-0711-6 (PMC5591526; doi:10.1186/s12882-017-0711-6)
Supplement: Supplementary file 1 — Intra-examiner reliability. Tables showing the results of the intra-examiner reliability testing for dichotomous and continuous variables. (PDF 352 kb) [file 12882_2017_711_MOESM1_ESM.pdf]

Factors associated with foot ulceration and amputation in adults on dialysis: a cross-sectional observational study

Michelle R Kaminski, Anita Raspovic, Lawrence P McMahon, Katrina A Lambert, Bircan Erbas, Peter F Mount, Peter G Kerr, Karl B Landorf

---

Additional File 1 Intra-examiner reliability

Reliability statistics for intra-examiner comparisons for the monofilament test and palpation of pedal pulses are shown in Additional Table 1. Linear weighted kappa values equaled 1.0 (95% confidence interval, CI, 1.0 to 1.0) for all variables, indicating excellent levels of agreement. Absolute percentage of agreement ranged from 95 to 100%.

Additional Table 1 Intra-examiner reliability for dichotomous data

	Linear weighted kappa	95% CI	Absolute % agreement	Interpretation*
**Monofilament**				
Left	1.00	1.00 to 1.00	95	Excellent agreement
Right	1.00	1.00 to 1.00	95	Excellent agreement
**Pedal pulses**				
Left	1.00	1.00 to 1.00	95	Excellent agreement
Right	1.00	1.00 to 1.00	100	Excellent agreement

CI = Confidence interval. *Weighted kappa values >0.8 represent excellent agreement, >0.6 substantial levels of agreement, 0.4 to 0.6 moderate agreement and <0.4 poor to fair agreement.

Reliability statistics for intra-examiner comparisons for the vibration perception threshold, ankle-brachial pressure index, toe-brachial pressure index and range of motion of the first metatarsophalangeal joint were found to have good reliability and are shown in Additional Table 2. The 95% limits of agreement ranged between -4.21 to 4.72. Intra-class correlations coefficients (ICCs) and corresponding 95% confidence intervals (CIs) for the continuous variables (left and right, respectively) were: 0.99 (95% CI, 0.98 to 1.00) and 0.99 (95% CI, 0.97 to 1.00) for vibration perception threshold, 0.95 (0.87 to 0.98) and 0.91 (0.79 to 0.96) for ankle brachial pressure index, 0.87 (0.70 to 0.95) and 0.88 (0.72 to 0.95) for toe-brachial

pressure index, and 0.99 (0.97 to 0.99) and 0.99 (0.98 to 1.00) for first metatarsophalangeal joint range of motion.

Additional Table 2 Intra-examiner reliability for continuous data

	ICC	95% CI	95% LOA	Interpretation*
**Vibration perception threshold**				
Left	0.99	0.98 to 1.00	-2.89 to 3.51	Good reliability
Right	0.99	0.97 to 1.00	-4.21 to 4.72	Good reliability
**Ankle-brachial pressure index**				
Left	0.95	0.87 to 0.98	-0.16 to 0.18	Good reliability
Right	0.91	0.79 to 0.96	-0.17 to 0.24	Good reliability
**Toe-brachial pressure index**				
Left	0.87	0.70 to 0.95	-0.18 to 0.15	Good reliability
Right	0.88	0.72 to 0.95	-0.17 to 0.14	Good reliability
**Range of motion (1st MTPJ)**				
Left	0.99	0.97 to 0.99	-2.57 to 3.50	Good reliability
Right	0.99	0.98 to 1.00	-2.53 to 2.33	Good reliability

ICC = Intra-class correlation coefficient. CI = Confidence interval. LOA = Limits of agreement. *ICC values >0.75 indicate good reliability, ICCs ranging from 0.50 to 0.75 imply moderate reliability and ICCs <0.50 suggest poor reliability. MTPJ = Metatarsophalangeal joint.
