# Supplementary material for: Factors associated with foot ulceration and amputation in adults on dialysis: a cross-sectional observational study
Source: BMC Nephrol. 2017 Sep 8;18:293. doi: 10.1186/s12882-017-0711-6 (PMC5591526; doi:10.1186/s12882-017-0711-6)
Supplement: Supplementary file 2 — Risk factors and potential confounding variables. A table showing the risk factors and potential confounding variables that were considered in the regression models. (PDF 361 kb) [file 12882_2017_711_MOESM2_ESM.pdf]

Factors associated with foot ulceration and amputation in adults on dialysis: a cross-sectional observational study

Michelle R Kaminski, Anita Raspovic, Lawrence P McMahon, Katrina A Lambert, Bircan Erbas, Peter F Mount, Peter G Kerr, Karl B Landorf

Additional File 2 Risk factors and potential confounding variables

**Continuous variables**	**Categorical variables**
Participant characteristics<br>• **Age**<br>• **Body mass index**<br>Health-related quality of life (SF-36v2®)<br>• Physical Component Score<br>• Mental Component Score<br>Comorbidities<br>• **Duration of diabetes**<br>Dialysis-related variables<br>• **Duration of dialysis**<br>Laboratory blood tests<br>• C-reactive protein<br>• Serum albumin<br>• Total calcium<br>• Serum phosphate<br>• Parathyroid hormone<br>• Glycated hemoglobin (HbA1c)<br>• Hemoglobin<br>Other<br>• Peak plantar pressures	Participant characteristics<br>• **Male sex**<br>• **Current smoker**<br>• **Living alone**<br>Comorbidities<br>• **Diabetes mellitus**<br>• **Retinopathy**<br>• **Peripheral neuropathy**<br>• **Peripheral arterial disease**<br>• **Arterial calcification**<br>• **Hypertension**<br>• **Dyslipidemia**<br>• **Ischemic heart disease**<br>• **Congestive cardiac failure**<br>• **Cerebrovascular disease**<br>• Osteoarthritis<br>• Inflammatory arthritis<br>Lower extremity complications<br>• **Previous foot ulceration**<br>• **Current foot ulcer present at baseline**<br>• **Previous lower extremity amputation**<br>Other<br>• Reduced range of motion of the 1st metatarsophalangeal joint<br>• **Foot deformity**<br>• **Inappropriate/ill-fitting footwear**<br>• Skin pathology<br>• Nail pathology<br>• Poor foot health care behaviors<br>• Regular podiatry attendance

Potential confounding variables that were considered in the regression models are boldface.

The above table is sourced from:
Kaminski MR, Raspovic A, McMahon LP, Erbas B, Landorf KB. Risk factors for foot ulceration in adults with end-stage renal disease on dialysis: study protocol for a prospective observational cohort study. J Foot Ankle Res. 2015 Sep; 8:53.

© 2015 Kaminski et al. **Open Access** This article is distributed under the terms of the Creative Commons Attribution 4.0 International License (http://creativecommons.org/licenses/by/4.0/), which permits unrestricted use, distribution, and reproduction in any medium, provided you give appropriate credit to the original author(s) and the source, provide a link to the Creative Commons license, and indicate if changes were made. The Creative Commons Public Domain Dedication waiver (http://creativecommons.org/publicdomain/zero/1.0/) applies to the data made available in this article, unless otherwise stated.
