# Supplementary material for: Factors associated with foot ulceration and amputation in adults on dialysis: a cross-sectional observational study
Source: BMC Nephrol. 2017 Sep 8;18:293. doi: 10.1186/s12882-017-0711-6 (PMC5591526; doi:10.1186/s12882-017-0711-6)
Supplement: Supplementary file 3 — Characteristics of current foot ulcers and amputations. Tables showing the characteristics of current foot ulcers and amputations at baseline. (PDF 527 kb) [file 12882_2017_711_MOESM3_ESM.pdf]

Factors associated with foot ulceration and amputation in adults on dialysis: a cross-sectional observational study

Michelle R Kaminski, Anita Raspovic, Lawrence P McMahon, Katrina A Lambert, Bircan Erbas, Peter F Mount, Peter G Kerr, Karl B Landorf

---

Additional File 3 Characteristics of current foot ulcers and amputations

Additional Table 1 Characteristics of current foot ulcers – data are total number of foot ulcers (%), unless otherwise specified

	Total foot ulcers	Left foot ulcers	Right foot ulcers
Total number of foot ulcers	68 (100.0)	28 (41.2)	40 (58.8)
Number of foot ulcers/participant			
Mean (SD), Range	1.51 (0.89), 1.0 to 5.0	1.27 (0.55), 1.0 to 3.0	1.38 (0.86), 1.0 to 5.0
Median (IQR)	1.0 (1.0 to 2.0)	1.0 (1.0 to 1.0)	1.0 (1.0 to 1.0)
Location			
Toes (dorsal, lateral or medial)	36 (52.9)	15 (53.6)	21 (52.5)
Plantar toes, forefoot and midfoot	16 (23.5)	5 (17.9)	11 (27.5)
Dorsal foot	8 (11.8)	5 (17.9)	3 (7.5)
Heel	8 (11.8)	3 (10.7)	5 (12.5)
Type			
Neuropathic	8 (11.8)	2 (7.1)	6 (15.0)
Neuro-ischemic	47 (69.1)	18 (64.3)	29 (72.5)
Ischemic	4 (5.9)	4 (14.3)	0 (0)
Other	9 (13.2)	4 (14.3)	5 (12.5)
Median duration (IQR), *months*	3.0 (1.2 to 6.0)	2.5 (1.0 to 5.0)	3.0 (1.8 to 6.5)

SD = Standard deviation. IQR = Interquartile range.

Additional Table 2 Characteristics of amputations – data are total number of amputations (%), unless otherwise specified

	Total amputations	Left amputations	Right amputations
Total number of amputations	79 (100.0)	49 (62.0)	30 (38.0)
Number of amputations/participant			
Mean (SD), Range	1.72 (0.94), 1.0 to 4.0	1.48 (0.76), 1.0 to 4.0	1.25 (0.68), 1.0 to 4.0
Median (IQR)	1.0 (1.0 to 2.0)	1.0 (1.0 to 2.0)	1.0 (1.0 to 1.0)
Minor	66 (83.5)	40 (81.6)	26 (86.7)
Toe	54 (81.8)	36 (90.0)	18 (69.2)
Partial toe	1 (1.5)	0 (0)	1 (3.8)
Metatarsal	1 (1.5)	0 (0)	1 (3.8)
Transmetatarsal	10 (15.2)	4 (10.0)	6 (23.1)
Major	13 (16.5)	9 (18.4)	4 (13.3)
Below knee amputation	13 (100)	9 (100)	4 (100)
Above knee amputation	0 (0)	0 (0)	0 (0)

SD = Standard deviation. IQR = Interquartile range.
