# Supplementary material for: Factors associated with foot ulceration and amputation in adults on dialysis: a cross-sectional observational study
Source: BMC Nephrol. 2017 Sep 8;18:293. doi: 10.1186/s12882-017-0711-6 (PMC5591526; doi:10.1186/s12882-017-0711-6)
Supplement: Supplementary file 4 — Comparisons between participants with and without diabetes. Tables showing comparisons between participants with and without diabetes for the presence and absence of foot complications. (PDF 690 kb) [file 12882_2017_711_MOESM4_ESM.pdf]

Factors associated with foot ulceration and amputation in adults on dialysis: a cross-sectional observational study

Michelle R Kaminski, Anita Raspovic, Lawrence P McMahon, Katrina A Lambert, Bircan Erbas, Peter F Mount, Peter G Kerr, Karl B Landorf

---

Additional File 4 Comparisons between participants with and without diabetes

Additional Table 1 Foot complications in participants *with* current foot ulceration and/or amputation – data are *n* (%), unless otherwise stated

	Foot ulceration				Amputation			
	**Total (n = 45)**	**Diabetes (n = 39)**	**No diabetes (n = 6)**	***P*-value***	**Total (n = 46)**	**Diabetes (n = 43)**	**No diabetes (n = 3)**	***P*-value***
Previous foot ulceration	32 (71.1)	30 (76.9)	2 (33.3)	0.087	44 (95.7)	41 (95.3)	3 (100)	1.00
Current foot ulceration	45 (100.0)	39 (100.0)	6 (100.0)	N/A†	26 (56.5)	24 (55.8)	1 (33.3)	1.00
Previous lower extremity amputation	26 (57.8)	24 (61.5)	2 (33.3)	0.39	46 (100)	43 (100)	3 (100)	N/A†
Minor	22 (48.9)	20 (51.3)	2 (33.3)	0.70	39 (84.8)	36 (83.7)	3 (100)	1.00
Major	8 (17.8)	8 (20.5)	0 (0)	0.52	12 (26.1)	12 (27.9)	0 (0)	0.70
Combination	4 (8.9)	4 (10.3)	0 (0)	0.96	5 (10.9)	5 (11.6)	0 (0)	1.00
Peripheral neuropathy	43 (95.6)	38 (97.4)	5 (83.3)	0.62	44 (95.7)	42 (97.7)	2 (66.7)	0.28
Peripheral arterial disease	42 (93.3)	36 (92.3)	6 (100.0)	1.00	39 (84.8)	37 (86.0)	2 (66.7)	0.94
Arterial calcification	20 (44.4)	18 (46.2)	2 (33.3)	0.88	21 (45.7)	20 (46.5)	1 (33.3)	1.00
Foot deformity	39 (86.7)	33 (84.6)	6 (100.0)	0.70	42 (91.3)	39 (90.7)	3 (100)	1.00
Limited range of motion of first MTPJ‡	39 (86.7)	34 (87.2)	5 (83.3)	N/A†	38 (82.6)	36 (83.7)	2 (66.7)	N/A†
Mean peak plantar pressures (SD), *kg/cm²*‡								
Total left foot	2.05 (0.53)	2.08 (0.52)	1.84 (0.61)	0.57	2.06 (0.37)	2.07 (0.37)	1.70§	0.33
Total right foot	2.13 (0.55)	2.15 (0.56)	1.99 (0.53)	0.67	2.13 (0.50)	2.18 (0.46)	1.04§	0.023*
Skin pathology	42 (93.3)	37 (94.9)	5 (83.3)	0.86	43 (93.5)	40 (93.0)	3 (100)	1.00
Nail pathology	37 (82.2)	32 (82.1)	5 (83.3)	1.00	34 (73.9)	33 (76.7)	1 (33.3)	0.33
Inappropriate footwear	25 (55.6)	20 (51.3)	5 (83.3)	0.30	21 (45.7)	20 (46.5)	1 (33.3)	1.00

Poor foot-health care	10 (22.2)	9 (23.1)	1 (16.7)	1.00	7 (15.2)	7 (16.3)	0 (0)	1.00
Podiatry attendance, *last 12 months*	36 (80.0)	31 (79.5)	5 (83.3)	1.00	36 (78.3)	34 (79.1)	2 (66.7)	1.00

*Significant difference between 'diabetes' and 'no diabetes' groups, $p < 0.05$. N/A = Not applicable. †Unable to calculate p-value due to limited number of cases. MTPJ = Metatarsophalangeal joint. ‡For foot ulceration, maximum missing data were for left peak plantar pressure involving 17 participants overall (37.8%) – missing data were for limited range of motion of first MTPJ (left, n = 16; right, n = 9) and peak plantar pressures (left, n = 17; right, n = 16). For amputation, maximum missing data were for left limited range of motion of first MTPJ involving 25 participants overall (54.3%) – missing data were for limited range of motion of first MTPJ (left, n = 25; right, n = 14) and peak plantar pressures (left, n = 22; right, n = 21). §Exact value recoded as only one participant.

Additional Table 2 Foot complications in participants *without* current foot ulceration and/or amputation – data are *n* (%), unless otherwise stated

	No foot ulceration				No amputation			
	**Total (n = 405)**	**Diabetes (n = 187)**	**No diabetes (n = 218)**	***P*-value***	**Total (n = 404)**	**Diabetes (n = 183)**	**No diabetes (n = 221)**	***P*-value***
Previous foot ulceration	65 (16.0)	45 (24.1)	20 (9.2)	<0.001*	53 (13.1)	34 (18.6)	19 (8.6)	0.005*
Current foot ulceration	N/A	N/A	N/A	N/A	19 (4.7)	15 (8.2)	4 (1.8)	0.005*
Previous lower extremity amputation	20 (4.9)	19 (10.2)	1 (0.5)	<0.001*	N/A	N/A	N/A	N/A
Minor	17 (4.2)	16 (8.6)	1 (0.5)	<0.001*				
Major	4 (1.0)	4 (2.1)	0 (0)	0.096				
Combination	1 (0.2)	1 (0.5)	0 (0)	0.94				
Peripheral neuropathy	185 (45.7)	111 (59.4)	74 (33.9)	<0.001*	184 (45.5)	107 (58.5)	77 (34.8)	<0.001*
Peripheral arterial disease	194 (47.9)	113 (60.4)	81 (37.2)	<0.001*	197 (48.8)	112 (61.2)	85 (38.5)	<0.001*
Arterial calcification	164 (40.5)	87 (46.5)	77 (35.3)	0.029*	163 (40.3)	85 (46.4)	78 (35.3)	0.030*
Foot deformity	302 (74.6)	141 (75.4)	161 (73.9)	0.81	299 (74.0)	135 (73.8)	164 (74.2)	1.00
Limited range of motion of first MTPJ†	382 (94.3)	184 (98.4)	198 (90.8)	<0.001*	383 (94.8)	182 (99.5)	201 (91.0)	<0.001*
Mean peak plantar pressures (SD), *kg/cm²*†								
Total left foot	1.84 (0.57)	1.92 (0.58)	1.77 (0.55)	0.012*	1.84 (0.58)	1.93 (0.60)	1.77 (0.55)	0.012*
Total right foot	1.85 (0.61)	1.93 (0.56)	1.79 (0.64)	0.035*	1.86 (0.61)	1.93 (0.57)	1.80 (0.64)	0.048*
Skin pathology	353 (87.2)	165 (88.2)	188 (86.2)	0.65	352 (87.1)	162 (88.5)	190 (86.0)	0.54
Nail pathology	282 (69.6)	144 (77.0)	138 (63.3)	0.004*	285 (70.5)	143 (78.1)	142 (64.3)	0.003*
Inappropriate footwear	272 (67.2)	131 (70.1)	141 (64.7)	0.30	276 (68.3)	131 (71.6)	145 (65.6)	0.24
Poor foot-health care	126 (31.1)	39 (20.9)	87 (39.9)	<0.001*	129 (31.9)	41 (22.4)	88 (39.8)	<0.001*
Podiatry attendance, *last 12 months*	187 (46.2)	122 (65.2)	65 (29.8)	<0.001*	187 (46.3)	119 (65.0)	68 (30.8)	<0.001*

*Significant difference between 'diabetes' and 'no diabetes' groups, $p < 0.05$. N/A = Not applicable. MTPJ = Metatarsophalangeal joint. †For foot ulceration, maximum missing data were for peak plantar pressures involving 39 participants overall (9.6%) – missing data were for limited range of motion of first MTPJ (left, n = 9; right, n = 6) and peak plantar pressures (left, n = 39; right, n = 39). For amputation, maximum missing data were for peak plantar pressures involving 34 participants overall (8.4%) – missing data were for limited range of motion of first MTPJ (right, n = 1) and peak plantar pressures (left, n = 34; right, n = 34).
