# Supplementary material for: Factors associated with foot ulceration and amputation in adults on dialysis: a cross-sectional observational study
Source: BMC Nephrol. 2017 Sep 8;18:293. doi: 10.1186/s12882-017-0711-6 (PMC5591526; doi:10.1186/s12882-017-0711-6)
Supplement: Supplementary file 5 — Individual health-related quality of life, foot assessment, and foot-health care behavior results for participants with and without foot ulceration and/or amputation. Tables showing comparisons between participants with and without foot ulceration and/or amputation for health-related quality of life, foot assessment, and foot-health care behavior variables – data are presented in its entirety before the data were categorized for statistical analysis. (PDF 732 kb) [file 12882_2017_711_MOESM5_ESM.pdf]

Factors associated with foot ulceration and amputation in adults on dialysis: a cross-sectional observational study

Michelle R Kaminski, Anita Raspovic, Lawrence P McMahon, Katrina A Lambert, Bircan Erbas, Peter F Mount, Peter G Kerr, Karl B Landorf

---

Additional File 5 Individual health-related quality of life, foot assessment, and foot-health care behavior results for participants *with* and *without* foot ulceration and/or amputation

The following tables included in this additional file present the data in its entirety before the data were categorized for statistical analysis (presented in Table 2 of journal article).

Additional Table 1 Health-related quality of life (SF-36v2®) – data are mean (SD)

		**Total (n = 450)**	**Foot ulceration**		**Lower extremity amputation**	
			**Yes (n = 45)**	**No (n = 405)**	**Yes (n = 46)**	**No (n = 404)**
Physical component score	Physical functioning	36.00 (11.34)	30.28 (10.47)	36.64 (11.27)	29.50 (9.68)	36.75 (11.29)
	Role physical	40.44 (11.64)	36.40 (11.54)	40.89 (11.59)	37.73 (11.87)	40.75 (11.60)
	Bodily pain	46.39 (13.40)	41.35 (13.43)	46.95 (13.29)	43.87 (15.18)	46.68 (13.17)
	General health	39.87 (10.90)	37.41 (11.21)	40.15 (10.85)	35.40 (11.39)	40.38 (10.74)
Mental component score	Vitality	43.47 (10.79)	40.65 (11.07)	43.78 (10.73)	41.10 (10.59)	43.74 (10.80)
	Social functioning	42.40 (14.03)	39.51 (15.17)	42.72 (13.88)	36.74 (16.21)	43.04 (13.63)
	Role emotional	45.19 (11.76)	41.78 (15.13)	45.57 (11.28)	43.30 (14.02)	45.40 (11.47)
	Mental health	48.58 (10.83)	46.74 (11.33)	48.79 (10.77)	47.17 (12.07)	48.74 (10.69)

SF-36v2® = Short-Form-36 version 2.0.

Additional Table 2 Foot assessment – data are *n* (%), unless otherwise stated

		Total (n = 450)	Foot ulceration		Lower extremity amputation	
			Yes (n = 45)	No (n = 405)	Yes (n = 46)	No (n = 404)
Peripheral neuropathy	*Mean vibration perception threshold (SD), V*					
	Left foot*	20.0 (11.9)	31.1 (11.5)	19.1 (11.5)	30.2 (11.0)	19.3 (11.7)
	Right foot*	19.1 (11.6)	30.0 (10.8)	18.3 (11.3)	28.8 (10.6)	18.4 (11.4)
	Vibration >50 V left or right foot	37 (8.2)	18 (40.0)	19 (4.7)	19 (41.3)	18 (4.5)
	Protective sensation					
	Left foot*					
	0/3	78 (17.3)	24 (53.3)	54 (13.3)	28 (60.9)	50 (12.4)
	1/3	34 (7.6)	5 (11.1)	29 (7.2)	3 (6.5)	31 (7.7)
	2/3	49 (10.9)	4 (8.9)	45 (11.1)	4 (8.7)	45 (11.1)
	3/3	280 (62.2)	6 (13.3)	274 (67.7)	2 (4.3)	278 (68.8)
	Right foot*					
	0/3	77 (17.1)	27 (60.0)	50 (12.3)	30 (65.2)	47 (11.6)
	1/3	31 (6.9)	5 (11.1)	26 (6.4)	2 (4.3)	29 (7.2)
	2/3	61 (13.6)	6 (13.3)	55 (13.6)	8 (17.4)	53 (13.1)
	3/3	277 (61.6)	5 (11.1)	272 (67.2)	2 (4.3)	275 (68.1)
Peripheral arterial disease	*Pedal pulses*					
	Left foot*					
	0/2	59 (13.1)	20 (44.4)	39 (9.6)	16 (34.8)	43 (10.6)
	1/2	87 (19.3)	13 (28.9)	74 (18.3)	10 (21.7)	77 (19.1)
	2/2	295 (65.6)	6 (13.3)	289 (71.4)	11 (23.9)	284 (70.3)
	Right foot*					
	0/2	60 (13.3)	24 (53.3)	36 (8.9)	18 (39.1)	42 (10.4)
	1/2	84 (18.7)	11 (24.4)	73 (18.0)	9 (19.6)	75 (18.6)
	2/2	302 (67.1)	8 (17.8)	294 (72.6)	15 (32.6)	287 (71.0)
	Mean ankle-brachial pressure index (SD)					
	Left foot*	1.12 (0.27)	0.90 (0.33)	1.13 (0.25)	1.00 (0.41)	1.12 (0.25)
	Right foot*	1.15 (0.28)	1.11 (0.37)	1.14 (0.27)	1.11 (0.38)	1.15 (0.27)
	Mean toe-brachial pressure index (SD)					
	Left foot*	0.70 (0.26)	0.56 (0.31)	0.71 (0.26)	0.62 (0.30)	0.70 (0.26)
	Right foot*	0.72 (0.25)	0.52 (0.21)	0.73 (0.25)	0.57 (0.22)	0.73 (0.25)
Foot deformity	Hammer/claw toes	212 (47.1)	27 (60.0)	185 (45.7)	28 (60.9)	184 (45.5)
	Hallux abducto valgus	265 (58.9)	26 (57.8)	239 (59.0)	21 (45.7)	244 (60.4)
	Stage 1	27 (10.2)	0 (0)	27 (11.3)	1 (4.8)	26 (10.7)

	Stage 2	132 (49.8)	15 (57.7)	117 (49.0)	16 (76.2)	116 (47.5)
	Stage 3	88 (33.2)	7 (26.9)	81 (33.9)	2 (9.5)	86 (35.2)
	Stage 4	18 (6.8)	4 (15.4)	14 (5.9)	2 (9.5)	16 (6.5)
	Bony prominences	50 (11.1)	14 (31.1)	36 (8.9)	16 (34.8)	34 (8.4)
	Charcot neuroarthropathy	16 (3.6)	5 (11.1)	11 (2.7)	11 (23.9)	5 (1.2)
	Other	122 (27.1)	23 (51.1)	99 (24.4)	28 (60.9)	94 (23.3)
Limited range of motion of first MTPJ	*Mean dorsiflexion of first MTPJ (SD), degrees*					
	Left foot*	36.0 (14.0)	28.9 (11.2)	36.5 (14.1)	26.4 (9.9)	36.5 (14.0)
	Right foot*	34.6 (14.8)	26.3 (12.3)	35.4 (14.7)	24.2 (11.7)	35.5 (14.7)
Peak plantar pressures	*Mean total left foot (SD), kg/cm²**	1.85 (0.57)	2.05 (0.53)	1.84 (0.57)	2.06 (0.37)	1.84 (0.58)
	*Mean total right foot (SD), kg/cm²**	1.87 (0.61)	2.13 (0.55)	1.85 (0.61)	2.13 (0.50)	1.86 (0.61)
Footwear	*Length*					
	Good	336 (74.7)	36 (80.0)	300 (74.1)	35 (76.1)	301 (74.5)
	Too short	80 (17.8)	4 (8.9)	76 (18.8)	6 (13.0)	74 (18.3)
	Too long	34 (7.6)	5 (11.1)	29 (7.2)	5 (10.9)	29 (7.2)
	Width					
	Good	344 (76.4)	39 (86.7)	305 (75.3)	39 (84.8)	305 (75.5)
	Too narrow	105 (23.3)	6 (13.3)	99 (24.4)	7 (15.2)	98 (24.3)
	Too wide	1 (0.2)	0 (0)	1 (0.2)	0 (0)	1 (0.2)
	Depth					
	Good	336 (74.7)	36 (80.0)	300 (74.1)	40 (87.0)	296 (73.3)
	Too shallow	114 (25.3)	9 (20.0)	105 (25.9)	6 (13.0)	108 (26.7)
	Fixation					
	None	202 (44.9)	14 (31.1)	188 (46.4)	11 (23.9)	191 (47.3)
	Laces	145 (32.2)	11 (24.4)	134 (33.1)	16 (34.8)	129 (31.9)
	Velcro	76 (16.9)	19 (42.2)	57 (14.1)	17 (37.0)	59 (14.6)
	Straps/buckles	19 (4.2)	0 (0)	19 (4.7)	2 (4.3)	17 (4.2)
	Zips	8 (1.8)	1 (2.2)	7 (1.7)	0 (0)	8 (2.0)
	Forefoot sole flexion point					
	At level of MTPJs	406 (90.2)	38 (84.4)	368 (90.9)	41 (89.1)	365 (90.3)
	Proximal to first MTPJ	24 (5.3)	4 (8.9)	20 (4.9)	2 (4.3)	22 (5.4)
	Distal to first MTPJ	20 (4.4)	3 (6.7)	17 (4.2)	3 (6.5)	17 (4.2)
	Heel height					
	0-2.5 cm	426 (94.7)	43 (95.6)	383 (94.6)	45 (97.8)	381 (94.3)
	2.6-5.0 cm	14 (3.1)	0 (0)	14 (3.5)	1 (2.2)	13 (3.2)
	>5.0 cm	10 (2.2)	2 (4.4)	8 (2.0)	0 (0)	10 (2.5)
	Materials					
	Leather	128 (28.4)	15 (33.3)	113 (27.9)	15 (32.6)	113 (28.0)

	Synthetic	265 (58.9)	23 (51.1)	242 (59.8)	25 (54.3)	240 (59.4)
	Mesh	54 (12.0)	5 (11.1)	49 (12.1)	5 (10.9)	49 (12.1)
	Other	3 (0.7)	2 (4.4)	1 (0.2)	1 (2.2)	2 (0.5)
	Footwear style					
	Walking shoe	68 (15.1)	3 (6.7)	65 (16.0)	4 (8.7)	64 (15.8)
	Boot	13 (2.9)	1 (2.2)	12 (3.0)	2 (4.3)	11 (2.7)
	Slipper	22 (4.9)	6 (13.3)	16 (4.0)	4 (8.7)	18 (4.5)
	Sandal	31 (6.9)	1 (2.2)	30 (7.4)	2 (4.3)	29 (7.2)
	Athletic shoe	61 (13.6)	5 (11.1)	56 (13.8)	8 (17.4)	53 (13.1)
	Ugg-boot	11 (2.4)	1 (2.2)	10 (2.5)	2 (4.3)	9 (2.2)
	Backless slipper	6 (1.3)	1 (2.2)	5 (1.2)	0 (0)	6 (1.5)
	Surgical/bespoke	16 (3.6)	12 (26.7)	4 (1.0)	10 (21.7)	6 (1.5)
	Oxford shoe	42 (9.3)	6 (13.3)	36 (8.9)	6 (13.0)	36 (8.9)
	High heel	2 (0.4)	1 (2.2)	1 (0.2)	0 (0)	2 (0.5)
	Court shoe	58 (12.9)	1 (2.2)	57 (14.1)	0 (0)	58 (14.4)
	Moccasin	7 (1.6)	2 (4.4)	5 (1.2)	1 (2.2)	6 (1.5)
	Thong/flip-flop	50 (11.1)	1 (2.2)	49 (12.1)	1 (2.2)	49 (12.1)
	Mule	61 (13.6)	3 (6.7)	58 (14.3)	4 (8.7)	57 (14.1)
	Other	2 (0.4)	1 (2.2)	1 (0.2)	2 (4.3)	0 (0)
	Age of shoe					
	0-6 months	126 (28.0)	21 (46.7)	105 (25.9)	20 (43.5)	106 (26.2)
	6-12 months	27 (6.0)	4 (8.9)	23 (5.7)	3 (6.5)	24 (5.9)
	>12 months	297 (66.0)	20 (44.4)	277 (68.4)	23 (50.0)	274 (67.8)
Skin pathology	Hyperkeratosis	307 (68.2)	35 (77.8)	272 (67.2)	37 (80.4)	270 (66.8)
	Heloma dura	109 (24.2)	7 (15.6)	102 (25.2)	6 (13.0)	103 (25.5)
	Uremic pruritus	106 (23.6)	10 (22.2)	96 (23.7)	5 (10.9)	101 (25.0)
	Xerosis	202 (44.9)	31 (68.9)	171 (42.2)	28 (60.9)	174 (43.1)
	Mild					
	1	115 (56.9)	11 (35.5)	104 (60.8)	12 (42.9)	103 (59.2)
	2	57 (28.2)	9 (29.0)	48 (28.1)	9 (32.1)	48 (27.6)
	Moderate					
	3	21 (10.4)	8 (25.8)	13 (7.6)	5 (17.9)	16 (9.2)
	4	8 (4.0)	3 (9.7)	5 (2.9)	2 (7.1)	6 (3.4)
	Severe					
	5	1 (0.5)	0 (0)	1 (0.6)	0 (0)	1 (0.6)
	6	0 (0)	0 (0)	0 (0)	0 (0)	0 (0)
	Calciphylaxis	5 (1.1)	1 (2.2)	4 (1.0)	1 (2.2)	4 (1.0)
	Other	55 (12.2)	6 (13.3)	49 (12.1)	6 (13.0)	49 (12.1)

Nail pathology	Half-and-half nail	79 (17.6)	9 (20.0)	70 (17.3)	5 (10.9)	74 (18.3)
	Absent lunula	121 (26.9)	8 (17.8)	113 (27.9)	9 (19.6)	112 (27.7)
	Onychomycosis	101 (22.4)	17 (37.8)	84 (20.7)	15 (32.6)	86 (21.3)
	Onychocryptosis	3 (0.7)	0 (0)	3 (0.7)	0 (0)	3 (0.7)
	Onychauxis	121 (26.9)	18 (40.0)	103 (25.4)	19 (41.3)	102 (25.2)
	Other	10 (2.2)	0 (0)	10 (2.5)	0 (0)	10 (2.5)

SD = Standard deviation. V = Volts. *Maximum missing data were for ankle-brachial pressure index involving 79 participants missing overall (17.6%). Missing data were for vibration perception threshold (left, n = 36; right, n = 33), protective sensation (left, n = 9; right, n = 4), pedal pulses (left, n = 9; right, n = 4), ankle-brachial pressure index (n = 79), toe-brachial pressure index (left, n = 27; right, n = 26), limited range of motion of first MTPJ (left, n = 25; right, n = 15) and peak plantar pressures (left, n = 56; right, n = 55). MTPJ = Metatarsophalangeal joint.

Additional Table 3 Foot-health care behaviors – data are *n* (%)

	Total (n = 450)	Foot ulceration		Lower extremity amputation	
		Yes (n = 45)	No (n = 405)	Yes (n = 46)	No (n = 404)
Question 1. Do you inspect your feet daily?					
Yes	133 (29.6)	17 (37.8)	116 (28.6)	18 (39.1)	115 (28.5)
No	317 (70.4)	28 (62.2)	289 (71.4)	28 (60.9)	289 (71.5)
Question 2. Do you avoid walking barefoot?					
Yes	231 (51.3)	30 (66.7)	201 (49.6)	32 (69.6)	199 (49.3)
No	219 (48.7)	15 (33.3)	204 (50.4)	14 (30.4)	205 (50.7)
Question 3. Are you able to reach your feet?					
Yes	299 (66.4)	22 (48.9)	277 (68.4)	25 (54.3)	274 (67.8)
No	151 (33.6)	23 (51.1)	128 (31.6)	21 (45.7)	130 (32.2)
Question 4. Do you treat your own nails and skin lesions? (e.g. calluses or corns)					
Yes	205 (45.6)	5 (11.1)	200 (49.4)	7 (15.2)	198 (49.0)
No	36 (8.0)	4 (8.9)	32 (7.9)	2 (4.3)	34 (8.4)
Podiatrist	209 (46.4)	36 (80.0)	173 (42.7)	37 (80.4)	172 (42.6)
Question 5. Have you ever seen a podiatrist before?					
Yes	278 (61.8)	39 (86.7)	239 (59.0)	43 (93.5)	235 (58.2)
No	172 (38.2)	6 (13.3)	166 (41.0)	3 (6.5)	169 (41.8)
