# Supplementary material for: Factors associated with foot ulceration and amputation in adults on dialysis: a cross-sectional observational study
Source: BMC Nephrol. 2017 Sep 8;18:293. doi: 10.1186/s12882-017-0711-6 (PMC5591526; doi:10.1186/s12882-017-0711-6)
Supplement: Supplementary file 6 — Univariate analyses for factors associated with foot ulceration and lower extremity amputation. Tables showing the results of the univariate analyses for factors associated with foot ulceration and lower extremity amputation. (PDF 711 kb) [file 12882_2017_711_MOESM6_ESM.pdf]

Factors associated with foot ulceration and amputation in adults on dialysis: a cross-sectional observational study

Michelle R Kaminski, Anita Raspovic, Lawrence P McMahon, Katrina A Lambert, Bircan Erbas, Peter F Mount, Peter G Kerr, Karl B Landorf

---

Additional File 6 Univariate analyses for factors associated with foot ulceration and lower extremity amputation

Additional Table 1 Univariate analysis for factors associated with *foot ulceration* – data are *n* (%), unless otherwise stated

	**Foot ulceration**		**Univariate logistic regression analysis**		
**Risk Factor**	**Yes**	**No**	**OR**	**95% CI**	***P*-value***
n	45	405			
Mean age (SD), *years*	69.0 (10.3)	67.3 (13.5)	1.01	0.99 to 1.04	0.43
Male sex	36 (80.0)	255 (63.0)	2.35	1.10 to 5.02	0.027*
Mean body mass index (SD), *kg/m²*	29.1 (6.5)	28.1 (6.6)	1.02	0.98 to 1.07	0.30
Current smoking	5 (11.1)	49 (12.1)	0.91	0.34 to 2.41	0.85
Living alone	6 (13.3)	69 (17.0)	0.75	0.31 to 1.84	0.53
Median duration of dialysis (IQR), *months*	37.5 (20.0 to 64.1)	36.8 (16.5 to 71.8)	1.00	1.00 to 1.00	0.88
Diabetes mellitus	39 (86.7)	187 (46.2)	7.58	3.14 to 18.30	<0.001*
Mean diabetes duration (SD), *months*	348.8 (167.6)	237.0 (142.3)	1.01	1.00 to 1.01	<0.001*
Retinopathy	28 (62.2)	104 (25.7)	0.21	0.11 to 0.40	<0.001*
Peripheral neuropathy	43 (95.6)	185 (45.7)	0.04	0.01 to 0.16	<0.001*
Peripheral arterial disease	42 (93.3)	194 (47.9)	15.23	4.64 to 49.92	<0.001*
Arterial calcification	20 (44.4)	164 (40.5)	1.18	0.63 to 2.19	0.61
Hypertension (requiring medication)	36 (80.0)	324 (80.0)	1.00	0.46 to 2.16	1.00
Dyslipidemia	38 (84.4)	263 (64.9)	2.93	1.28 to 6.73	0.011*
Ischemic heart disease	36 (80.0)	227 (56.0)	3.14	1.47 to 6.68	0.003*
Congestive cardiac failure	18 (40.0)	104 (25.7)	1.93	1.02 to 3.65	0.043*
Cerebrovascular disease	19 (42.2)	85 (21.0)	2.75	1.45 to 5.21	0.002*
Osteoarthritis	17 (37.8)	175 (43.2)	0.80	0.42 to 1.50	0.49
Inflammatory arthritis	14 (31.1)	169 (41.7)	0.63	0.33 to 1.22	0.172
Median C-reactive protein (SD), *mg/L†*	10.00 (4.82 to 43.83)	6.95 (2.67 to 18.08)	1.01	1.00 to 1.02	0.058
Mean serum albumin (SD), *g/L*	31.12 (4.76)	34.01 (3.74)	0.84	0.78 to 0.90	<0.001*
Mean total calcium (SD), *mmol/L*	2.15 (0.13)	2.20 (0.14)	0.06	0.01 to 0.53	0.011*
Mean phosphate (SD), *mmol/L*	1.64 (0.39)	1.54 (0.38)	1.81	0.86 to 3.82	0.120
Median parathyroid hormone (SD), *pmol/L*	28.03 (20.70 to 43.02)	29.77 (17.38 to 46.25)	0.99	0.98 to 1.01	0.36
Mean glycated hemoglobin (SD), *%†*	6.89 (1.24)	6.05 (1.29)	1.51	1.22 to 1.86	<0.001*
Mean hemoglobin (SD), *g/L*	107.56 (10.62)	110.02 (15.22)	0.99	0.97 to 1.01	0.30
Mean SF-36v2® PCS (SD)<br>Mean SF-36v2® MCS (SD)	33.08 (8.92)<br>47.08 (13.96)	38.71 (10.74)<br>48.71 (11.09)	0.95<br>0.99	0.92 to 0.98<br>0.96 to 1.01	0.001*<br>0.36
Previous foot ulceration	32 (71.1)	65 (16.0)	12.88	6.41 to 25.85	<0.001*
Previous amputation	26 (57.8)	20 (4.9)	26.34	12.53 to 55.38	<0.001*
Foot deformity	39 (86.7)	302 (74.6)	2.22	0.91 to 5.39	0.079

Limited range of motion of first MTPJ†‡	39 (86.7)	382 (94.3)	N/A	N/A	N/A
Mean peak plantar pressure (SD), *kg/cm²*†<br>Total left foot<br>Total right foot	 <br>2.05 (0.53)<br>2.13 (0.55)	 <br>1.84 (0.57)<br>1.85 (0.61)	 <br>1.69<br>1.72	 <br>0.98 to 2.89<br>1.08 to 2.75	 <br>0.057<br>0.023*
Skin pathology	42 (93.3)	353 (87.2)	2.06	0.62 to 6.90	0.24
Nail pathology	37 (82.2)	282 (69.6)	2.02	0.91 to 4.46	0.083
Inappropriate/ill-fitting footwear	25 (55.6)	272 (67.2)	0.61	0.33 to 1.14	0.122
Poor foot health care	10 (22.2)	126 (31.1)	0.63	0.30 to 1.32	0.22
Podiatry attendance, *last 12 months*	36 (80.0)	187 (46.2)	4.66	2.19 to 9.93	<0.001*

OR = Odds ratio. CI = Confidence interval. *Significant association, $p < 0.05$. SD = Standard deviation. IQR = Interquartile range. †Maximum missing data were for left peak plantar pressure involving 56 participants overall (12.4%). Missing data were for glycated hemoglobin (n = 39), C-reactive protein (n = 3), limited range of motion of first MTPJ (left, n = 25; right, n = 15) and peak plantar pressures (left, n = 56; right, n = 55). SF-36v2® = Short-Form-36 Version 2. PCS = Physical component score. MCS = Mental component score. MTPJ = Metatarsophalangeal joint. ‡As limited first MTPJ range of motion was present in the majority of the sample and in all participants with foot ulceration, this variable was removed from the analysis. N/A = Not applicable.

Additional Table 2 Univariate analysis for factors associated with *lower extremity amputation* – data are *n* (%), unless otherwise stated

	**Lower extremity amputation**		**Univariate logistic regression analysis**		
**Risk Factor**	**Yes**	**No**	**OR**	**95% CI**	***P*-value***
n	46	404			
Mean age (SD), *years*	65.4 (11.23)	67.7 (13.4)	0.99	0.97 to 1.01	0.25
Male sex	37 (80.4)	254 (62.9)	2.43	1.14 to 5.17	0.021*
Mean body mass index (SD), *kg/m²*	30.4 (6.3)	27.9 (6.6)	1.05	1.01 to 1.10	0.015*
Current smoking	5 (10.9)	49 (12.1)	1.13	0.43 to 3.00	0.80
Living alone	10 (21.7)	65 (16.1)	1.45	0.69 to 3.06	0.33
Median duration of dialysis (IQR), *months*	38.3 (17.7 to 72.6)	36.6 (16.6 to 69.5)	1.00	1.00 to 1.01	0.92
Diabetes mellitus	43 (93.5)	183 (45.3)	17.31	5.28 to 56.71	<0.001*
Mean diabetes duration (SD), *months*	301.8 (163.1)	245.6 (148.5)	1.00	1.00 to 1.00	0.032*
Retinopathy	32 (69.6)	100 (24.8)	6.95	3.57 to 13.55	<0.001*
Peripheral neuropathy	44 (95.7)	184 (45.5)	26.30	6.29 to 109.98	<0.001*
Peripheral arterial disease	39 (84.8)	197 (48.8)	5.85	2.56 to 13.40	<0.001*
Arterial calcification	21 (45.7)	163 (40.3)	1.24	0.67 to 2.29	0.49
Hypertension (requiring medication)	40 (87.0)	320 (79.2)	1.75	0.72 to 4.27	0.22
Dyslipidemia	39 (84.8)	262 (64.9)	3.02	1.32 to 6.93	0.009*
Ischemic heart disease	33 (71.7)	230 (56.9)	1.92	0.98 to 3.76	0.057
Congestive cardiac failure	15 (32.6)	107 (26.5)	1.34	0.70 to 2.59	0.38
Cerebrovascular disease	15 (32.6)	89 (22.0)	1.71	0.89 to 3.31	0.110
Osteoarthritis	15 (32.6)	177 (43.8)	0.62	0.33 to 1.19	0.148
Inflammatory arthritis	13 (28.3)	170 (42.1)	0.54	0.28 to 1.06	0.074
Median C-reactive protein (SD), *mg/L*†	10.89 (4.44 to 36.88)	6.90 (2.67 to 18.50)	1.01	1.00 to 1.02	0.007*
Mean serum albumin (SD), *g/L*	32.50 (4.32)	33.86 (3.88)	0.92	0.85 to 0.99	0.026*
Mean total calcium (SD), *mmol/L*	2.21 (0.12)	2.20 (0.14)	1.55	0.17 to 14.62	0.70
Mean phosphate (SD), *mmol/L*	1.59 (0.41)	1.55 (0.38)	1.30	0.60 to 2.81	0.50
Median parathyroid hormone (SD), *pmol/L*	28.52 (21.56 to 50.24)	29.65 (17.38 to 45.43)	1.00	0.99 to 1.01	0.77
Mean glycated hemoglobin (SD), *%*†	6.98 (1.36)	6.03 (1.27)	1.58	1.29 to 1.95	<0.001*
Mean hemoglobin (SD), *g/L*	107.56 (10.62)	110.02 (15.22)	0.99	0.97 to 1.01	0.30
Mean SF-36v2® PCS (SD)	33.16 (9.93)	38.71 (10.65)	0.95	0.93 to 0.98	0.001*
Mean SF-36v2® MCS (SD)	47.11 (13.56)	48.71 (11.14)	0.99	0.96 to 1.01	0.37
Previous foot ulceration	44 (95.7)	53 (13.1)	145.70	34.31 to 618.77	<0.001*
Current foot ulceration	26 (56.5)	19 (4.7)	26.34	12.53 to 55.38	<0.001*
Foot deformity	42 (91.3)	299 (74.0)	3.69	1.29 to 10.53	0.015*
Limited range of motion of first MTPJ†‡	38 (82.6)	383 (94.8)	N/A	N/A	N/A
Mean peak plantar pressure (SD), *kg/cm²*†					
Total left foot	2.06 (0.37)	1.84 (0.58)	1.68	0.95 to 2.98	0.075
Total right foot	2.13 (0.50)	1.86 (0.61)	1.70	1.04 to 2.78	0.036*
Skin pathology	43 (93.5)	352 (87.1)	2.12	0.63 to 7.07	0.22
Nail pathology	34 (73.9)	285 (70.5)	1.18	0.59 to 2.36	0.63
Inappropriate/ill-fitting footwear	21 (45.7)	276 (68.3)	0.39	0.21 to 0.72	0.003*
Poor foot health care	7 (15.2)	129 (31.9)	0.38	0.17 to 0.88	0.024*
Podiatry attendance, *last 12 months*	36 (78.3)	187 (46.3)	4.18	2.02 to 8.65	<0.001*

OR = Odds ratio. CI = Confidence interval. *Significant association, $p < 0.05$. SD = Standard deviation. IQR = Interquartile range. †Maximum missing data were for left peak plantar pressure involving 56 participants overall (12.4%). Missing data were for glycated hemoglobin (n = 39), C-reactive protein (n = 3), limited range of motion of first MTPJ (left, n = 25; right, n = 15) and peak plantar pressures (left, n = 56; right, n = 55). SF-36v2® = Short-Form-36 Version 2. PCS = Physical component score. MCS = Mental component score. MTPJ = Metatarsophalangeal joint. ‡As limited first MTPJ range of motion was present in the majority of the sample and in all participants with amputation, this variable was removed from the analysis. N/A = Not applicable.
